# Hepatic Artery Aneurysm/Pseudoaneurysm: An Unusual Cause of Upper Gastrointestinal (UGI) Bleeding and Biliary Obstruction Further Complicated by Glue Dislodgement Leading to Biliary Obstruction

**DOI:** 10.7759/cureus.46031

**Published:** 2023-09-26

**Authors:** Umar M Khan, Ahmed S Hussain, Ahmad Khalaf, Christine Joerres, Matthew Potter

**Affiliations:** 1 Internal Medicine, The Royal London Hospital, London, GBR; 2 Gastroenterology, The Royal London Hospital, London, GBR; 3 Gastroenterology, Whipps Cross University Hospital, London, GBR

**Keywords:** visceral artery pseudoaneurysms, glue migration post angioemobilzation, transarterial embolisation, upper gi bleeds, hepatic artery aneurysms

## Abstract

Hepatic artery aneurysms (HAA) and pseudoaneurysms are rare vascular abnormalities, that can lead to significant morbidity and mortality if left untreated. We present a case report of a 78-year-old lady with a hepatic artery aneurysm who initially presented with upper gastrointestinal bleeding (UGIB) and biliary obstruction and was treated by trans-arterial embolization. Recovery was complicated by glue embolisation leading to obstructive jaundice and biliary sepsis. This case highlights the importance of having a high index of suspicion for HAA and pseudoaneurysm when initial investigations including oesophago gastro duodenoscopy (OGD) are negative. Although rare, glue embolization should be considered in patients who present with obstructive jaundice and abdominal pain.

## Introduction

Hepatic artery aneurysms (HAA) and pseudoaneurysms are uncommon vascular abnormalities characterized by abnormal dilatation of hepatic arteries. They are often found incidentally and up to 80% are pseudoaneurysms [1]. The most common risk factors for the development of hepatic artery aneurysms are hypertension, diabetes, vascular disease, smoking, and autoimmune diseases. The most common site for developing HAA is the common hepatic artery followed by the right hepatic artery. They can lead to significant morbidity and mortality especially if they rupture, leading to hemodynamic compromise from intra-abdominal bleeding. HAAs can present with symptoms of epigastric pain, pulsatile mass in the right upper quadrant, biliary obstruction, nausea/vomiting, melaena, or haematemesis. CT abdomen and CT angiography remain the best imaging modalities to diagnose hepatic artery aneurysms/pseudoaneurysms. Here, we present a case report of HAA which presented with upper gastrointestinal bleeding (UGIB) and biliary obstruction that represented a diagnostic and therapeutic challenge.

## Case presentation

A 78-year-old lady presented to the emergency department with a suspected UGIB. She complained of coffee-ground vomiting and melaena. Co-morbidities included hypertension, chronic type A aortic dissection under routine surveillance, mechanical aortic valve replacement, and root repair. She was haemodynamically compromised with an initial blood pressure of 94/58 mmHg and a heart rate of 91 bpm. She also had a transient episode of unresponsiveness which was attributed to hypotension.

In the laboratory investigations, we observed a notable decline in hemoglobin levels, starting at 103 g/L, then dropping to 96 g/L, and ultimately reaching 63 g/L. Simultaneously, there was an increase in urea levels, rising from 5 mmol/L to 9.9 mmol/L, alongside an initial INR of 2.5. It's important to note that the patient was under a steady regimen of warfarin (5 mg once daily), aiming for a target range of three to four, attributable to her mechanical aortic valve.

**Figure 1:**
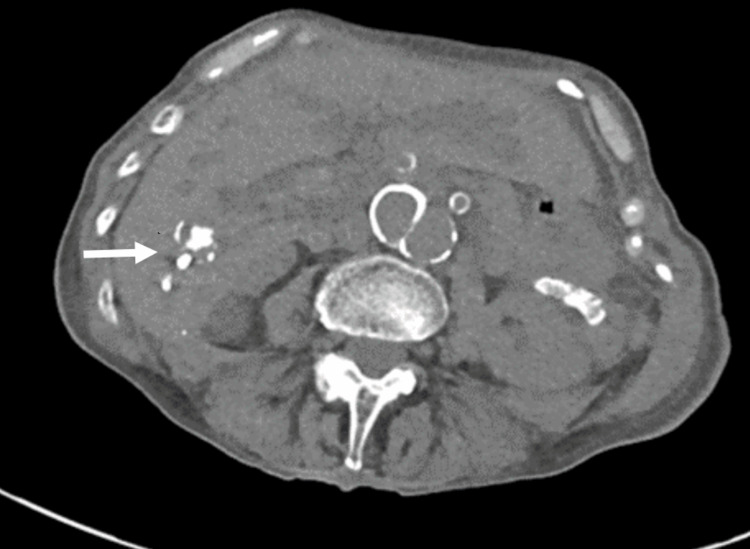
CT AP showing bleeding from the aneurysm causing haemobilia

**Figure 2:**
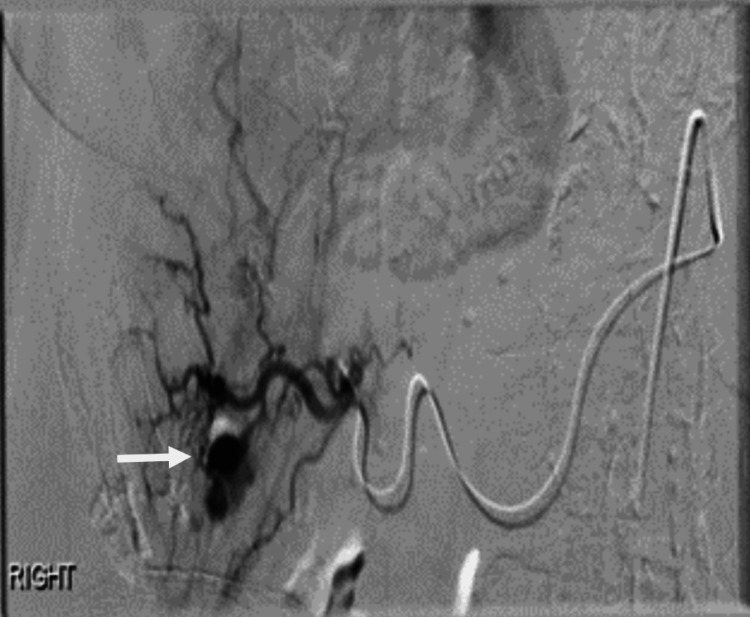
Angiogram demonstrating aneurysm from branch of right hepatic artery

She was initially resuscitated with 2 units of packed red cells because of a significant hemoglobin drop and hemodynamic compromise with symptoms. The reversal of anticoagulation was achieved with vitamin K and octaplex (prothrombin complex concentrate). An urgent oesophago gastro duodenoscopy (OGD) failed to identify an active bleeding point and only showed non-erosive gastritis. Subsequently, a video capsule endoscopy was performed which showed a normal appearance of the small bowel, and colonoscopy showed smooth muscle hypertrophy in the sigmoid colon and diverticular disease. A CT scan of the abdomen and pelvis was conducted to investigate the underlying cause of unexplained bleeding, abnormal liver function test results, and a significant decrease in hemoglobin levels. CT showed hyperdensity in the right lobe of the liver (Figure 1) and biliary dilatation. CT angiogram was performed due to ongoing bleeding and persistent hemoglobin drops, which demonstrated a 6mm right hepatic artery aneurysm within the right liver lobe (Figure 2). Bleeding from the aneurysm through the arterio-biliary fistula leads to biliary obstruction and common bile duct (CBD) dilatation into the ampulla of Vater where it abruptly tapered to a normal caliber.

A comprehensive multidisciplinary team, comprising gastroenterologists, cardiologists, and hematologists, initially opted for a conservative management strategy. The primary focus was on effectively controlling gastrointestinal bleeding and concurrently reducing the International Normalized Ratio (INR) to a target range of 1.5-2.0. This approach aimed to mitigate the inherent bleeding risk associated with hepatic artery aneurysms (HAA), all the while carefully considering the delicate balance required to prevent thromboembolism in the presence of a mechanical valve.

**Figure 3:**
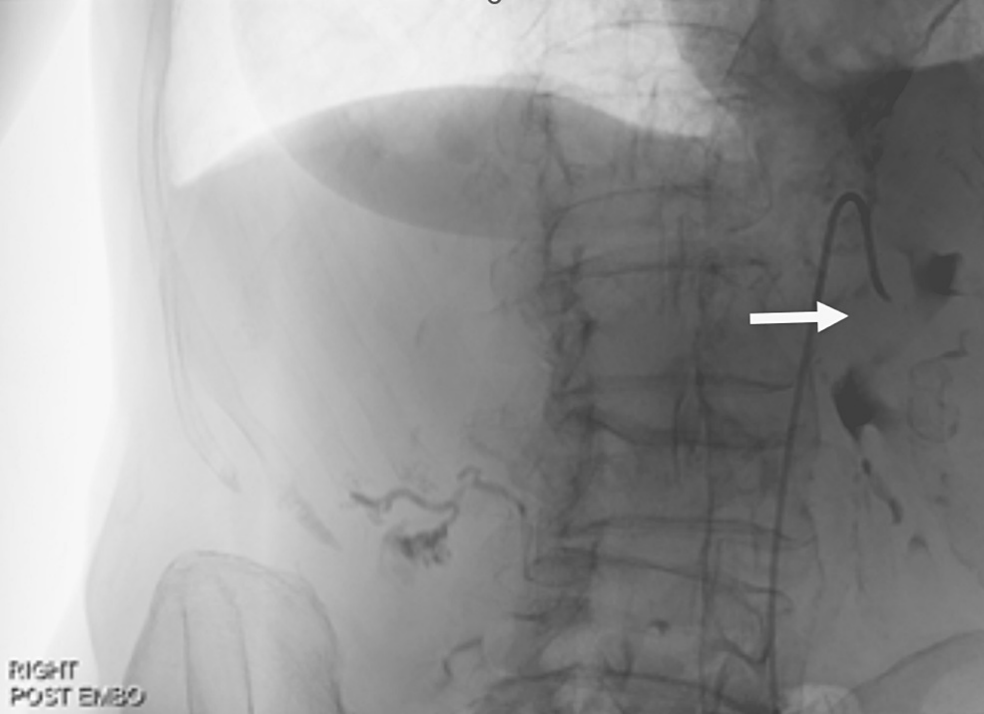
Showing hepatic artery aneurysms (HAA) post embolization

However, given the patient's recurrent admissions due to upper gastrointestinal bleeding (UGIB), the management approach evolved towards a more interventional strategy. She underwent HAA embolization using a combination of 1:1 glubran and lipidol (Figure 3). This intervention proved to be highly effective in addressing the aneurysm and resolving the bleeding issue.

In contrast to previous cases where hepatic artery aneurysms (HAA) were primarily managed through embolization or surgical interventions, the approach in this particular case adapted to the patient's complex medical background.

**Figure 4:**
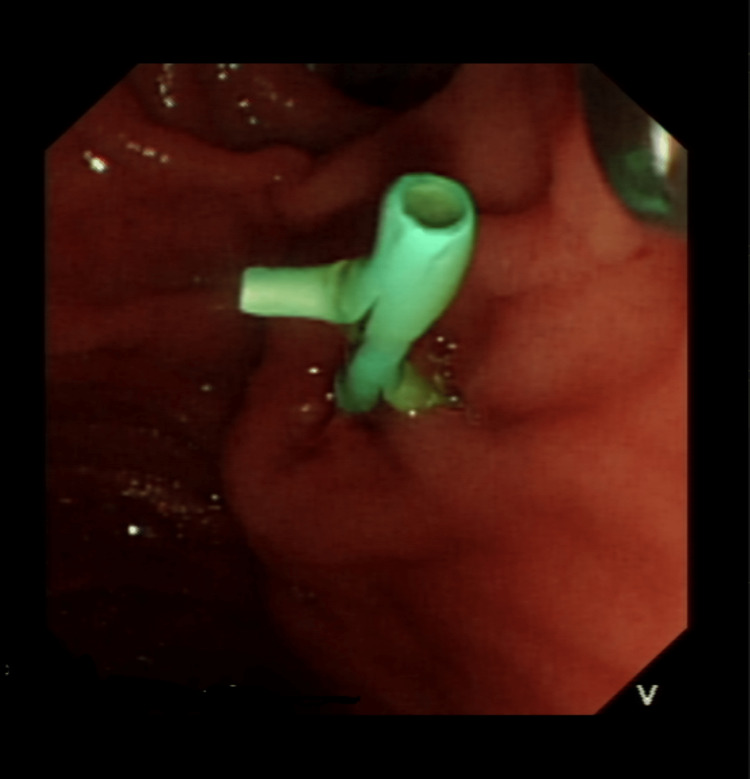
Pancreatic duct stent

**Figure 5:**
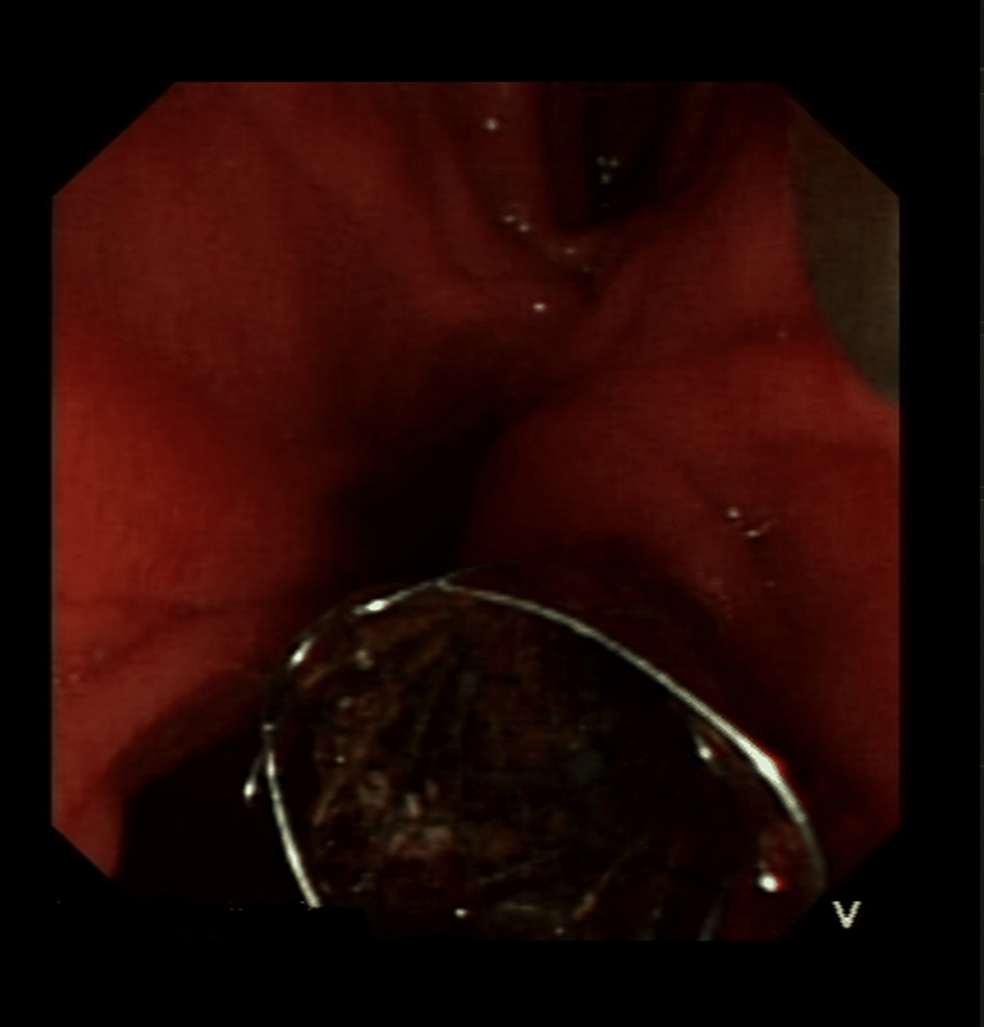
Showing fully covered self expanding metallic stent in CBD

**Figure 6:**
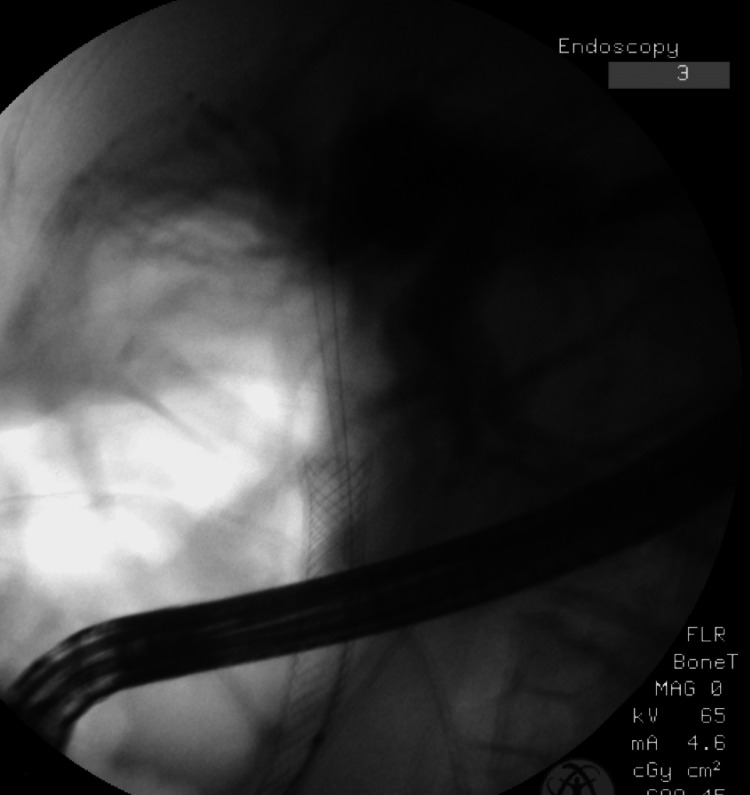
Shows guidewires in CBD with metallic stent already deployed

The patient was again readmitted a few days later with right upper quadrant pain, jaundice, melaena, and signs of biliary sepsis. CT abdomen/pelvis showed that there was a spillage of glue into the distal CBD causing biliary obstruction. She underwent successful endoscopic retrograde cholangiopancreatography with sphincterotomy, and placement of a 60mm covered metal stent placed in the CBD along with prophylactic stent placement in the pancreatic duct (Figures 4, 5, 6). Gradually her liver function tests and inflammatory markers improved (Table 1). She was discharged with close gastroenterology follow-up and bi-annual CT angiograms.

**Table 1:** Pre and post procedure lab values

	Pre procedure values	Post Procedure values
Bilirubin	153 micro mol/l	26 micro mol/l
ALT	67 unit/l	26 unit/l
ALP	742 unit/L	317 unit/l
CRP	92 mg/L	17 mg/L

## Discussion

This case highlights the importance of considering HAA/pseudoaneurysm as a potential cause of UGIB and biliary obstruction. Pseudoaneurysms are usually symptomatic whilst true HAAs are usually asymptomatic and often found incidentally. However, in up to 80% of cases, rupture can be the initial clinical manifestation associated with a mortality rate of 35-80% [2]. Proposed risk factors for HAAs include atherosclerosis, trauma, infection, vasculitis, connective tissue disorders, and iatrogenic causes, including post-liver transplantation [3,4]. The potential risk factors for developing HAA in her case were hypertension and vascular disease.

Clinical presentation can be varied including abdominal pain, a pulsatile mass in the right upper quadrant, nausea/vomiting, and haemobilia. Obstructive jaundice can occur due to external compression of the CBD or intraductal blood clots secondary to fistula formation between the pancreatic and biliary ducts [5,6]. The triad of jaundice, biliary colic, and gastrointestinal bleeding, as seen in our patients is seen in up to 25% of patients [7-9]. The lady in the case presentation had initially presented with lethargy, abdominal pain, multiple episodes of haematemesis, melaena, and then also developed biliary obstruction.

Ultrasound Doppler can be useful in determining hepatic flow and the dimensions of the aneurysm, however, it has limited sensitivity to detect small aneurysms [10]. US Doppler liver and portal vein were done in this case as well, which demonstrated Doppler flow at the superior aspect of the embolised pseudoaneurysm which was concerning for the persisting residual flow. CT angiography is the current investigation of choice for diagnosis due to its precision, lower complication rate, and wider availability, particularly in medical emergencies. Invasive angiography can also be considered [4,11].

Pseudoaneurysms should be repaired regardless of size due to the high bleeding risk. For true HAAs, treatment decisions are based on the size, location, rate of increase in size, and patient's overall health [4]. Endovascular embolization techniques, such as coil and glue, are usually the first-line approach. Open surgical options like aneurysmectomy may be considered in hemodynamically unstable patients or when endovascular intervention fails [3,4].

Embolization procedures have several indications, the most important being Hemorrhage Control followed by treatment of vascular malformations. The contraindications of the procedure are allergic reactions (to the contrast dye or embolic agents used in embolization), infections, impaired renal function, pregnancy, coagulation disorders, and poor vessel access.

Embolization may lead to various complications, such as impaired liver function and necrosis of the liver or gallbladder due to reduced blood supply, the formation of abscesses, and liver rupture. Erosion and migration of the embolic agent into the biliary tree to the best of our knowledge is very rare. In our case, glue migration may have been triggered by a small arterial-biliary fistula, initially resulting in haemobilia. Over time, this fistula could have expanded, leading to the erosion of the glue into the biliary tree. The presence of glue inside the biliary tree can cause obstructions and stone formation, necessitating its removal. To prevent such complications, it is crucial to avoid packing pseudo-aneurysmal sacs and to carefully consider the proximity of more central HAA to the bile ducts [12].

## Conclusions

Although not commonly encountered in clinical practice, HAAs and pseudoaneurysms should be considered in the differential diagnosis of UGIB and biliary obstruction, in the context of a normal OGD. Endovascular embolization can serve as a safe and effective treatment option for carefully selected patients. Glue embolization remains a rare complication and should be considered in patients who present with obstructive jaundice and abdominal pain.
